# Feasibility and Preferences to Adopt mHealth-Based Interventions for HIV Prevention Among High-Risk Groups: Cross-Sectional Study

**DOI:** 10.2196/81111

**Published:** 2026-03-12

**Authors:** Faizur Rehman, Muhammad Wasay Shahid, Mehran Riaz, Malik Muhammad Umair, Farah Azhar, Adeel Siddiqui, Ali Ahmed

**Affiliations:** 1 Department of Pharmacy Practice, Riphah Institute of Pharmaceutical Sciences, Riphah International University, Islamabad, Pakistan; 2 Common Management Unit, National Institute of Health, Islamabad, Pakistan; 3 Department of Pharmaceutics, Riphah Institute of Pharmaceutical Sciences, Riphah International University, Islamabad, Pakistan; 4 Department of Pharmacy, Shaukat Khanum Memorial Cancer Hospital and Research Center, Lahore, Pakistan; 5 Division of Infectious Diseases and Global Public Health, School of Medicine, University of California, San Diego, La Jolla, CA, 92093, United States, 1 619-953-9767

**Keywords:** mobile health, mHealth, mHealth apps, smartphone, mobile phone, HIV, HIV prevention, smartphone apps, health technology, key populations, marginalized communities, digital health

## Abstract

**Background:**

HIV disproportionately affects men who have sex with men (MSM), transgender individuals, and people who inject drugs, with 70.1% of cases in Pakistan linked to these groups, aggravated by stigma and legal constraints. Mobile health (mHealth) interventions offer the potential to enhance HIV prevention, yet their acceptability remains underexplored.

**Objective:**

This study aimed to assess the feasibility, willingness to use, and preferences for mHealth-based interventions designed to improve HIV knowledge, testing uptake, and risk behavior reduction among MSM, transgender individuals, and people who inject drugs in Pakistan.

**Methods:**

A cross-sectional survey was conducted from February 2025 to May 2025 with 210 participants, including MSM and transgender individuals, recruited through respondent-driven sampling in collaboration with community-based organizations and nongovernmental organizations, while people who inject drugs were recruited from the rehabilitation centers of trusted community-based organizations and nongovernmental organizations, to ensure privacy and reduce safety concerns. Participants completed a self-administered questionnaire covering demographics, HIV knowledge, access to and use of mobile technology, acceptability and preferences for mHealth interventions, and HIV-related risk behaviors.

**Results:**

The majority of participants owned smartphones (161/210, 76.7%) and had daily internet access (115/210, 54.8%), spending an average of 3.41 (SD 3.10) hours online daily. High willingness (182/210, 86.7%) to use a clinic-integrated mHealth app for HIV prevention was reported. Preferred app-delivered features included educational resources (85/210, 40.5%), daily medication reminders (108/210, 51.4%), weekly HIV prevention information (110/210, 52.4%), e-consultation (158/210, 75.2%), and mental health information (134/210, 63.8%). Smartphone ownership (odds ratio 4.14, 95% CI 1.41‐12.21; *P*=.009) and prior experience with an mHealth app (odds ratio 2.97, 95% CI 1.00‐8.81; *P*=.05) were significantly associated with willingness to adopt an mHealth app. While 80.5% (169/210) reported some knowledge of HIV, only 17.1% (29/169) rated their knowledge high, and 46.1% (97/210) were unaware of their HIV status. In terms of vulnerability, participants reported that injected drug use (122/210, 58.1%), experiences of violence (87/210, 41.4%), and police detention (128/210, 61%) were prevalent. In terms of risky sexual behaviors, nearly half engaged in transactional sex (104/210, 49.5%) or had multiple partners (101/210, 48.1%), while consistent condom use remained low (90/210, 42.9%).

**Conclusions:**

There is high acceptability and considerable potential for mHealth interventions to enhance HIV prevention efforts among MSM, transgender individuals, and people who inject drugs in Pakistan. Tailored, discreet, and comprehensive mHealth platforms that include educational content, medication reminders, e-consultation, and mental health support are warranted. Addressing criminalization and ensuring user privacy will be crucial for the successful design and implementation of these interventions. Future research should focus on implementation research to assess the real-world uptake of these findings.

## Introduction

Despite remarkable advancements in HIV care and prevention, the acquisition of HIV remains a significant public health issue globally [1]. As of 2023, approximately 39.9 million people were living with HIV worldwide, including 1.3 million who were newly diagnosed, around 0.45 million of these in Southeast Asia [2]. The epidemic still disproportionately impacts key populations: the HIV prevalence was 11 times higher among gay men and other men who have sex with men, 7 times higher among people who inject drugs, and 14 times higher among transgender individuals, in comparison to the general adult population [3]. Discrimination, stigmatization, and punitive laws targeting key populations are costing lives and preventing the world from achieving the agreed global 95-95-95 AIDS target: 95% know their HIV status, 95% diagnosed with HIV are on antiretroviral therapy (ART), and 95% of those on ART are virally suppressed. Structural barriers, including poverty, limited health care access, and legal and human rights challenges, raise the likelihood of HIV infection 35-fold for people who inject drugs, 30-fold for female sex workers, and 14-fold for transgender women compared to adults in the general population [4]. Globally, studies confirm that HIV incidence remains high among sex workers, especially in countries that criminalize sex work compared to their counterparts in countries where sex work is wholly or partially legalized [5].

Mobile health (mHealth), defined as the use of mobile technologies for health services, has emerged as a promising tool for improving global health care access and delivery, improving health outcomes, and promoting preventive health behaviors [1,6]. mHealth, particularly smartphone apps and messaging, holds great promise for HIV prevention, especially when linked to HIV testing and pre-exposure prophylaxis (PrEP). The World Health Organization (WHO) and the Joint United Nations Program on HIV/AIDS have endorsed the integration of mHealth in HIV care strategies due to its reach across low-, middle-, and high-income countries [7]. For example, a study from China found that men who have sex with men who received customized short-message interventions reported significantly lower rates of risky sexual activities like multiple sex partners and condomless anal sex than those who did not receive the intervention [8].

Pakistan, the world’s fifth-most populous country, has an estimated 0.33 million people living with HIV. According to the latest data, only 23% of people living with HIV know their HIV status; among them, 74% are on ART [9]. In the absence of recent national viral suppression data, estimates can be drawn from the 2023 Joint United Nations Program on HIV/AIDS report, which indicated that 34,000 individuals on ART have achieved viral suppression [2]. When compared with ART enrollment figures reported by the country’s National AIDS Control Program, this suggests an approximate viral load of 63% among people living with HIV on ART in Pakistan [9]. Pakistan continues to face significant funding constraints in its HIV/AIDS response. The reallocation of HIV/AIDS-related resources to address the COVID-19 pandemic has further strained an already under-resourced health system, leading to disruptions in essential services for sexual and gender diverse populations [10]. Notably, in 2025, the HIV/AIDS component implemented by the National AIDS Control Program in collaboration with the United Nations Development Program (UNDP) Pakistan has experienced a reduction exceeding US $4 million [11]. Pakistan is still falling short of achieving the previous 90-90-90 global target set for 2020, and remains far from the current 2025 goals [12,13]. The number of new HIV infections in Pakistan increased from 14,000 in 2010 to 25,000 in 2020 [14]. AIDS-related deaths in Pakistan surged from 1400 in 2010 to 8000 in 2020 [3]. The HIV epidemic in Pakistan has shown evidence of shifting from isolated key populations to spreading into broader sexual networks. In Pakistan, 70.1% of HIV cases belong to key groups: men who have sex with men (22%), people who inject drugs (38.4%), female sex workers (2.2%), and transgender individuals (7.5%) [15]. Legal provisions such as Section 377 of the Pakistan Penal Code further exacerbate stigma and limit access to essential HIV services [16]. A total of 190 million cellular mobile connections were active as early as 2025, equivalent to 75.2% of the total population, and over 45.7% were using the internet [17]. Recent studies in other Asian and low- and middle-income countries have shown strong willingness among men who have sex with men, transgender individuals, and people who inject drugs to engage with mHealth platforms for HIV-related services [1,6,18].

Despite this, data on the feasibility and acceptability of mHealth solutions in Pakistan remain sparse, particularly among the most at-risk communities. A few digital platforms, such as “Sehat Dost” UNDP with support from the Global Fund and “HIV/AIDS Pakistan” by the Association of People Living with HIV, offer HIV information, self-assessment tools, and self-testing kits [19]. However, these apps lack integration, user-centered features, and functionality typical of comprehensive mHealth solutions. To address this, this study explores the willingness, preferences, and feasibility of adopting mHealth-based interventions among men who have sex with men, transgender individuals, and people who inject drugs in Pakistan. It aims to generate evidence for designing effective, user-centered digital tools that have the potential to improve HIV knowledge, increase testing uptake, and reduce high-risk behaviors in marginalized communities.

## Methods

### Study Design and Setting

A cross-sectional study was conducted from February 2025 to May 2025, using the quantitative survey-based method to assess the willingness, preferences, and feasibility of mHealth interventions for HIV prevention among the key populations in Pakistan, with data collected through collaboration with community-based organizations (CBOs) and nongovernment organizations (NGOs). The STROBE (Strengthening the Reporting of Observational Studies in Epidemiology) guideline (Checklist 1) was followed to conduct this study.

### Sample Size

The minimum sample size for estimating the proportion willing to use mHealth was first calculated with the single-proportion formula (95% CI, 5% precision, and anticipated prevalence=50%), giving 384 individuals. Because the accessible sampling frame in our sites was approximately 500 individuals, we applied a finite-population correction [20], resulting in 218 individuals. Anticipating minor design clustering across the 3 key population strata, a design effect of 1.1 was used, yielding approximately 240 individuals. However, the final achieved sample size was limited to 210 due to practical constraints such as limited recruitment time and resource limitations. Furthermore, many individuals from marginalized populations were reluctant to participate because of fears related to stigma, confidentiality, and social consequences. Although the final sample size was slightly lower than the initial target, it does not meaningfully compromise the overall interpretability of our findings. A post hoc power check indicates that with a sample of 210 participants, the study still retains acceptable precision for estimating willingness proportions with approximately ±6% to 7% precision at a 95% CI [21,22].

### Definitions of Key Terms

#### Key Population

Groups who, due to specific higher-risk behaviors, are at increased risk of HIV, irrespective of the epidemic type or local context. Also, they often have legal and social issues related to their behaviors that increase their vulnerability to HIV. According to WHO guidelines, key populations relevant to this study include: (1) men who have sex with men, (2) people who inject drugs, and (3) transgender individuals [23].

#### Men Who Have Sex With Men

This group encompasses male individuals who engage in sexual activities with other male individuals, regardless of their sexual orientation or identity. The term is used to focus on behavior rather than identity, recognizing that not all men who have sex with men identify as gay or bisexual [24].

#### People Who Inject Drugs

Individuals who inject psychotropic (or psychoactive) substances for nonmedical purposes through intravenous, intramuscular, subcutaneous, or other injectable routes. People who inject drugs are at heightened risk of HIV due to practices such as sharing contaminated injection equipment. This definition also applies to people who inject other nonmedical substances.

#### Transgender Individuals

An umbrella term for individuals whose gender identity and expression do not conform to the norms and expectations traditionally associated with the sex assigned to them at birth; it includes people who are transsexual, transgender, or otherwise gender nonconforming. Transgender people may self-identify as transgender, female, male, a transgender woman, or a transgender man [25].

#### Study Participants and Procedures

Inclusion criteria were (1) being aged 18 years or older; (2) self-identified as men who have sex with men, transgender individuals, or people who inject drugs; (3) resident of Pakistan; and (4) being able to understand Urdu or English.

Other key populations, such as female sex workers and people in prisons, were not included due to legal and ethical considerations. Although multiple groups are criminalized under Section 377, female sex workers in Pakistan are subjected to stronger policies, social stigma, and the absence of secure community-based networks, which create substantial barriers to safe, confidential, and ethical recruitment [16,26]. These factors present significant barriers to safe recruitment and ethical engagement in research contexts involving these groups.

Respondents were recruited using respondent-driven sampling (RDS), which is a network-based sampling method commonly used to recruit the hard-to-reach population, and combined with peer-driven recruitment [27]. Initial respondent-driven sampling participants, also called seed participants, were selected upon recommendations of trusted NGOs and CBOs for men who have sex with men and transgender individuals. We selected a sample of 10 seed participants from each group to ensure diversity. Each seed who completed the interviewer-administered questionnaire received 5 recruitment coupons to invite their peers. In turn, the subsequent participants were also given 5 coupons to recruit more peers. Each coupon card contained a unique RDS number that allowed us to trace the peer recruitment chain and related study information. Coupon management software tracked, distributed, and redeemed coupons during the study, and a standard numbering system was implemented to monitor the recruiter-recruit relationship. Due to ethical and security concerns, people who inject drugs were recruited through convenience sampling from rehabilitation centers and harm reduction centers operated by trusted NGOs and CBOs. The participants were only recruited if they were stable, conscious, and could provide informed consent. This approach was adopted to ensure privacy, reduce participant risk, and allow for more secure and supportive recruitment and data collection. Data collection was conducted in the private room of the NGO or CBO to ensure participants’ trust and privacy. Individuals could choose whether to come during the working hours of the NGO or CBO or set an appointment by phone. Individuals who presented with a valid coupon underwent initial eligibility screening. If eligible, an Android tablet was provided to complete the survey questionnaire, which was hosted on Google Forms in a private room. Participants were allowed to self-identify their gender from a list of inclusive options or choose not to disclose, in line with ethical guidelines for gender identity data collection [28]. Each participant completed the questionnaire in approximately 20 minutes, while study staff waited outside to ensure privacy.

### Ethical Considerations

The ethical approval for this study was obtained from the research and ethics committee of Riphah Institute of Pharmaceutical Sciences, Riphah International University (reference: REC-RIPS/RARE/2025/16); all ethical guidelines for research involving human participants were followed. Before participating, all participants were verbally informed about the purpose of the study, the nature of their involvement, potential risks and benefits, and their rights as research participants. Informed written consent was secured from all participants before data collection. Moreover, all the participants were informed that participation in this study was voluntary and that they could withdraw at any time. All participants were assured that their data would remain confidential, and they were informed that their data would be used for publication purposes. Participants were invited to self-identify their gender using inclusive options, with a choice to opt out, ensuring respect for individual autonomy and alignment with ethical standards for gender identity data collection. All the study procedures adopted complied with the Principles of the Declaration of Helsinki, Good Clinical Practices, and the applicable laws and regulations of research involving human subjects in Pakistan. The SAGER (Sex and Gender Equity in Research) guidelines were followed throughout the study and can be found in Checklist 2. Participants received PKR 500 (US $2.77) for study participation and an additional PKR 200 (US $1.11) for each peer successfully recruited to the study, up to a maximum of 5 peers.

### Measures

#### Overview

After reviewing the literature, the questionnaire for this study was adopted from a validated questionnaire used in a similar mHealth study in Malaysia [27]. After carefully reviewing the questionnaire and discussing it with other team members and the principal investigator, we made some modifications. Some questions were removed from the questionnaire, keeping in mind the cultural and ethical standards of the country. The questionnaire was subsequently translated into Urdu, and HIV experts in Pakistan, along with the principal investigator, evaluated its content validity. The main scale achieved a Cronbach α of 0.75, indicating acceptable reliability for a formative exploratory study. The questionnaire used for the study can be found in Multimedia Appendix 1.

#### Participant Characteristics

We collected participant characteristics: type (men who have sex with men, transgender individuals, or people who inject drugs), sexual orientation, education, employment status, and ethnicity. We did not take provincial and residential information to ensure confidentiality and privacy.

#### Knowledge About HIV Prevention

In the first section, participants were asked, “Do you know about HIV care and prevention methods?” Those who responded “Yes” to the previous question were asked to rate their HIV knowledge on a scale from 1 (very low) to 5 (very high).

#### Access to and Frequency of Use of Communication Technology

Participants were asked if they had their own smartphone and daily internet access, and the number of hours spent on the internet each week. Participants’ use of smartphones for various internet-based activities (including social networking, sending or receiving emails, using websites, searching for health-related information, or using health-related apps) was assessed using a 5-point Likert scale (ranging from 1=“never” to 5=“daily”). The participants were then asked if they had used any health-related mHealth apps previously, and a follow-up on their experience using the mHealth app was assessed using the Likert scale (ranging from 1=“very dissatisfied” to 5=“very satisfied.”

#### Acceptability of mHealth App

Participants were asked about their willingness to use a clinic-integrated mHealth app for HIV prevention, and their willingness to use 5 mHealth-related features: medication reminders, HIV prevention information, e-consultation with the doctor, mental health information, and engagement in a virtual mental health group. The study also evaluated respondents’ preferred frequency (daily, weekly, or monthly) and mode of mHealth delivery (such as phone calls, SMS text messaging, or mobile apps). Participants were asked, “What feature would you consider most important in a clinic-integrated mHealth app for HIV prevention?,” followed by a question: “On a scale of 1 (not at all feasible) to 5 (extremely feasible), how feasible do you think it is to integrate mobile apps into an HIV prevention program?”

#### Risky Sexual and Drug-Related Behavior

Participants were asked information about their sexual behavior, including recent (past 6 mo) engagement in anal sex, sexual relationships with multiple partners, consistent condom use, and transactional sexual relationships. Additionally, participants were asked about injectable drug use at any point in their lives. Participants were asked if they had ever tested for HIV and their HIV status. Participants were then asked if they had ever been diagnosed with other sexually transmitted infections (STIs), and if they had ever used PrEP or postexposure prophylaxis.

### Data Analysis

We calculated the descriptive statistics for categorical variables, such as frequency and percentage, and mean and SD for the continuous variables. Cross-tabulation and chi-square tests were used to measure the association between the categorical variables. Bivariate and multivariate logistic regression analyses were conducted to identify the correlates of mHealth acceptance. Results were reported as odds ratio (OR) with 95% CI. Analyses were performed in SPSS (Statistical Package for the Social Sciences; version 27; IBM Corp) and RStudio (2025.05.0+496), an integrated development environment for R (version 4.5.0; R Foundation for Statistical Computing).

## Results

### Participants’ Characteristics

A total of 210 participants were included in the study (mean age of 29.55, SD 4.82 y). Of the 210 participants, 83 (39.5%) were people who inject drugs, 72 (34.3%) were transgender individuals, and 55 (26.2%) were men who have sex with men. Regarding sexual orientation, 84 (40%) identified as heterosexual, while 72 (34.3%) identified as homosexual. Most respondents were college graduates (n=88, 41.9%), while only a few reported having completed higher education (n=25, 11.9%). Employment status showed that half of the participants were unemployed (n=105, 50%), and a large proportion identified themselves as Muslims (n=159, 75.7%; Table 1).

**Table 1.** Demographic characteristics of the participants (N=210).

Demographic characteristics	Value, n (%)
Key population	
Men who have sex with men	55 (26.2)
Transgender individuals	72 (34.3)
People who inject drugs	83 (39.5)
Sexual orientation	
Homosexual	72 (34.3)
Heterosexual	84 (40)
Bisexual	53 (25.2)
Education	
Less than high school	53 (25.2)
High school	88 (41.9)
College or associate	44 (21)
Higher education	25 (11.9)
Employment status	
Unemployed	105 (50)
Part-time employed	67 (31.9)
Full-time employed	32 (15.2)
Ethnicity or race	
Muslim	159 (75.7)
Non-Muslim	51 (24.3)
Knowledge about HIV and its transmission	
Yes	169 (80.5)
No	41 (19.5)
HIV knowledge rating	
Low	88 (52.1)
Moderate	54 (31.9)
High	29 (17.1)

Overall, 169 out of 210 (80.5%) participants reported having some knowledge of HIV and its transmission, while 29 out of 210 (17.1%) demonstrated a high level of HIV-related knowledge. Men who have sex with men reported the highest proportion of strong HIV knowledge (23/55, 41.8%), followed by transgender individuals (18/72, 25%) and people who inject drugs (13/83, 15.6%).

### Access to and Frequency of Use of Mobile Technology

Of the 210 participants, 161 (76.7%) had smartphones, and 115 (54.8%) had daily access to the internet. The most common activities that participants used the internet on their smartphones for were online social networking apps like Facebook, WhatsApp, or TikTok; on average, they spent 3.41 (SD 3.10) hours per day on them. A total of 77 out of 210 (36.7%) participants used mobile apps several times a week, and 74 out of 210 (35.2%) used them daily. Additionally, 127 out of 210 (60.5%) of the participants stated that they had used an mHealth app, and 94 out of 210 (44.7%) were satisfied with their experience with the mHealth app. A significant association was found between the education levels and previous mHealth app experiences (*χ*²_4_=24.08; *P*<.001). Among those who reported using the app (127/210, 60.5%), 40 out of 127 (31.4%) were transgender individuals, 27 out of 127 (21.2%) were men who have sex with men, and 32 out of 127 (25.1%) had a high school level education, suggesting a positive association between education and mHealth app engagement.

### Acceptability of the mHealth App

Of the 210 participants, 182 (86.7%) expressed a willingness to use a clinic-integrated mHealth app for HIV prevention (Table 2). More than half (n=108, 51.5%) indicated interest in a daily medication reminder feature, while 83 (39.5%) reported no interest in this function. Among those interested in medication reminders, 89 of 130 (68.5%) preferred to receive notifications through the app.

**Table 2.** Acceptability of clinic-integrated mobile health app for HIV prevention (N=210).

Variables	Value, n (%)
Clinic-integrated mobile app for HIV prevention	
No	28 (13.3)
Yes	182 (86.7)
Frequency of medication reminder	
Daily	108 (51.4)
Monthly	3 (1.4)
Never	83 (39.5)
Weekly	16 (7.6)
Preferred mechanism of getting reminders	
App notifications	89 (68.5)
Phone calls	17 (13.1)
SMS text messaging	21 (16.2)
Websites	3 (2.3)
Frequency of HIV prevention information	
Daily	21 (10)
Monthly	49 (23.3)
Never	30 (14.3)
Weekly	110 (52.4)
Preferred mechanism of HIV prevention information	
Apps	77 (43)
Phone calls	56 (31.3)
SMS text messaging	39 (21.8)
Websites	7 (3.9)
E-consultation	
No	52 (24.8)
Yes	158 (75.2)
Preferred mechanism of e-consultation	
Apps	75 (48.1)
Phone calls	68 (43.6)
SMS text messaging	8 (5.1)
Websites	5 (3.2)
Mental health information	
No	76 (36.2)
Yes	134 (63.8)
Preferred mechanism for mental health information	
Apps	73 (52.9)
Phone calls	40 (29)
SMS text messaging	19 (13.8)
Websites	6 (4.3)
Most important feature	
Educational resources	85 (40.5)
HIV self-testing information	42 (20.2)
Secure messaging with health care providers	28 (13.5)
Appointment reminders	15 (7.5)

Regarding the dissemination of HIV prevention information, 110 out of 210 (52.4%) respondents preferred receiving updates on a weekly basis, followed by 49 out of 210 (23.3%) who favored monthly updates. Among these, 77 out of 180 (42.7%) indicated a preference for receiving such information via the app. Additionally, two-thirds of the participants (158/210, 75.2%) expressed interest in e-consultation services, with preferences split between app-based consultations (75/156, 48.1%) and phone calls (68/156, 43.6%). Furthermore, 134 out of 210 (63.8%) participants showed interest in receiving mental health information, with 73 out of 138 (52.9%) preferring to access this content through the app. Most participants (85/210, 40.5%) indicated that educational resources was the most important feature, followed by information on HIV self-testing (42/210, 20.2%) and secure messaging with health care providers (28/210, 13.5%). Furthermore, a total of 75 out of 210 (35.7%) participants reported moderate feasibility, and 61 out of 210 (29%) reported high feasibility for an mHealth app for HIV prevention.

There was a positive association between smartphone ownership, daily internet access, and interest in mHealth for HIV prevention; 99.1% (114/115) of them were willing to use an mHealth app for HIV prevention. A chi-square test indicated a statistically significant association between smartphone use, daily internet access, and willingness to use an mHealth app (*χ*²_2_=71.19; *P*<.001). The likelihood-ratio chi-square test confirmed this association (*χ*²_2_=63.35; *P*<.001). The Cramer *V* was 0.41, indicating a moderately strong association between internet access, smartphone ownership, and willingness to use a clinic-integrated mHealth app.

### HIV Testing, Prevention, and Risk Behaviors

Out of 210 participants, 141 (67.1%) participants had been tested for HIV, but only 111 of 208 (52.9%) respondents knew their current HIV status. HIV prevention services uptake was limited, with 105 (50%) reporting no use; PrEP (47/88, 53.4%) was the most common. Self-reported awareness of STI status was moderate, with 92 out of 210 (43.8%) reporting that they knew their previous STI status. Participants reported a substantial level of vulnerability: 87 out of 210 (41.4%) had experienced violence, 128 out of 210 (61%) had been detained by the police, and 122 out of 210 (58.1%) used injectable drugs. Risky sexual behaviors were frequent, 104 out of 210 (49.5%) engaged in transactional sex, and 101 out of 210 (48.1%) reported multiple partners, while consistent condom use remained low (90/210, 42.9%), though slightly higher among men who have sex with men and transgender individuals (Table 3). Cross-tabulation analysis showed differences in patterns across groups. PrEP users reported higher use of a health-related mobile app. When grouped by sexual orientation, bisexual participants reported the highest levels of risky sexual behavior, followed by homosexual participants, while heterosexual participants reported the lowest levels. Participants who used any HIV prevention service tended to report moderately risky sexual behaviors, whereas those who did not use prevention services were more likely to report the highest levels of risk.

**Table 3.** HIV testing, prevention, and risk behavior (N=210).

Variables	Value, n (%)	
HIV testing		
Yes	141 (67.1)	
No	69 (32.9)	
Frequency of HIV testing		
Regularly (every year)	52 (36.4)	
Occasionally (every few years)	42 (29.4)	
Rarely	49 (34.3)	
HIV status		
Negative	111 (52.9)	
Do not know	97 (46.1)	
HIV prevention services		
Yes	105 (50)	
No	105 (50)	
Prevention services		
PrEP^a^	47 (53.4)	
PEP^b^	3 (3.4)	
Event-driven PrEP	20 (22.7)	
Safe needle practice	14 (15.9)	
Previously diagnosed with STI^c^		
Yes	36 (17.1)	
No	56 (26.7)	
Do not know	118 (56.2)	
PrEP use		
Yes	90 (42.8)	
No	120 (57.2)	
Violence in the past		
Yes	87 (41.4)	
No	123 (58.6)	
Detained by the police		
Yes	128 (61)	
No	82 (39)	
Depressive symptoms		
Yes	129 (61.4)	
No	81 (38.6)	
Injected drugs		
Yes	122 (58.1)	
No	88 (41.9)	
Anal sex in past 6 months		
Yes	98 (46.7)	
No	112 (53.7)	
Multiple sex partners in the past 6 months		
Yes	101 (48.1)	
No	109 (51.9)	
Transactional sex in the past 6 months		
Yes	104 (49.5)	
No	106 (50.5)	
Condoms in sexual encounters in the past 6 months		
Yes	90 (42.9)	
No	120 (57.1)	

a PrEP: pre-exposure prophylaxis.

b PEP: postexposure prophylaxis.

c STI: sexually transmitted infection.

### Correlates of mHealth App Acceptance

Part-time workers and students were significantly less likely to express a willingness to adopt a clinic-integrated mHealth app for HIV prevention compared to unemployed participants. Specifically, part-time workers had 70% lower odds (OR 0.30, 95% CI 0.12‐0.77; *P*=.01), and students had 87% lower odds (OR 0.13, 95% CI 0.02‐0.98; *P*=.047) of being willing to use the app. In contrast, participants who owned a smartphone were over 4 times more likely to be willing to adopt the mHealth app (OR 4.14, 95% CI 1.41‐12.21; *P*=.009). Moreover, individuals with prior experience using a mHealth app had nearly 3 times greater odds of willingness to adopt the app (OR 2.97, 95% CI 1.00‐8.81; *P*=.05) compared to those with no prior mHealth exposure (Table 4).

**Table 4.** Correlates of mHealth app acceptance.

Variables	OR^a^ (95% CI)	*P* value
Intercept	0.54 (0.25‐1.21)	.14
Key populations (vs people who inject drugs)		
Men who have sex with men	1.38 (0.43‐4.49)	.57
Transgender	1.46 (0.58‐3.69)	.43
Education (vs low education)		
Higher education	2.33 (0.85‐6.43)	.10
Employment (vs unemployed)		
Full time	0.57 (0.09‐3.73)	.56
Part time	0.30 (0.12‐0.77)	.01
Student	0.13 (0.02‐0.98)	.047
HIV knowledge (vs no)		
Yes	2.05 (0.78‐5.39)	.15
Smartphone ownership (vs no)		
Yes	4.14 (1.41‐12.21)	.009
Daily internet access (vs no)		
Yes	1.07 (0.36‐3.17)	.91
mHealth^b^ app experience (vs no)		
Yes	2.97 (1.00‐8.81)	.05

a OR: odds ratio.

b mHealth: mobile health.

## Discussion

### Principal Findings

This study is the first in Pakistan to explore the feasibility and acceptability of mHealth interventions for HIV prevention among key populations, including men who have sex with men, transgender individuals, and people who inject drugs. The findings reveal a significant unmet need for accessible HIV prevention services, evidenced by high-risk behaviors and low engagement with existing methods. However, the widespread use of smartphones and social networking platforms presents a promising avenue for digital health solutions. Participants showed strong interest in a clinic-integrated mHealth app, favoring features like educational content, medication reminders, and e-consultation services.

Our findings identify several key features that prospective users desire in an mHealth intervention for HIV prevention. Specifically, participants expressed strong interest in receiving daily medication reminders, weekly prevention information, access to e-consultation with health care providers, and mental health resources. These preferences align with evidence from India and Nepal, where men who have sex with men and transgender individuals similarly valued medication and appointment reminders, HIV prevention information, and teleconsultation services. In the Nepali study, men who have sex with men also emphasized additional needs such as screening and monitoring illicit drug use, chemsex behavior, and ordering HIV-related supplies, reflecting broader expectations for comprehensive digital support. However, one notable difference is that most participants in Nepal preferred monthly messages and reminders, which contradicts our finding of strong interest in daily reminders and weekly prevention content. Preferences reported in India were more consistent with our results, as participants supported daily medication reminders and weekly HIV prevention information [1,29,30]. In our study, consistent condom use in the past 6 months was reported by 63.6% of men who have sex with men and 62.5% of transgender participants. These rates are considerably higher than those reported in Malaysia, where only 19% of men who have sex with men reported condom use, and moderately higher than in Bangladesh, where 48.6% of men who have sex with men reported consistent use [27,31]. However, these findings were comparable with those of Indonesia, where 76.97% reported protected sex in the past 6 months [32]. Limited access to HIV prevention information, weak social and health care systems, and criminalization of same-sex behaviors and cultural taboos in Muslim-majority settings such as Malaysia, Indonesia, and Bangladesh intensify privacy concerns and restrict access to condoms and prevention strategies. These constraints likely contribute to inconsistent condom use and reduced engagement with HIV prevention programs in these contexts [31,33,34]. mHealth programs have shown promise in improving condom use and mental health. In one example, a self-paced web-based intervention for Chinese men who have sex with men dating app users significantly reduced condomless anal sex and increased participants’ use of self-efficacy and positive attitudes towards condoms [35]. More broadly, digital interventions in the settings of low- and middle-income countries have generally been found to enhance sexual and reproductive health knowledge and attitudes, which support safer behavior [36]. Our study found that participants who reported using HIV prevention services such as PrEP were often involved in moderately high-risk activities, and this finding aligns with existing research showing that PrEP use can sometimes be associated with an increase in risky sexual behaviors and the incidence of bacterial STIs [37,38]. This may reflect risk compensation or that individuals with higher baseline risk are more likely to seek PrEP. However, this association is not universal; studies in South-East Asia showed that PrEP use does not always translate into risk compensation, and educational awareness may lead to less involvement in high-risk behavior [39,40]. These findings suggest that integrating HIV prevention, reproductive health information, and risk-reduction counseling into mHealth platforms may strengthen consistent condom use and reduce risky sexual practices.

Also, high rates of violence (41.4%) and police detention (61%) were observed among men who have sex with men and people who inject drugs. Research in the Middle East and North Africa, including countries such as Pakistan, Iran, Lebanon, and Ghana, highlighted a high level of violence, stigma, and discrimination among sexual and gender minorities, with police often implicated in discriminatory practices [41]. These legal and social pressures create significant barriers to accessing care, a finding that is consistent with qualitative research on the lived experiences of people with HIV in Pakistan who face pervasive stigma and structural challenges [13]. Studies from Malaysia and Iran reported that high stigma and criminalization towards men who have sex with men and transgender individuals increased the privacy concerns and limited the open health communication and emphasized mHealth as a discreet option [6,42]. Similarly, a cross-sectional survey conducted among Malaysian men who have sex with men reported that although 74.9% of participants expressed willingness to use a hypothetical HIV prevention app, concerns related to privacy and confidentiality significantly limited acceptability [43]. A Malaysian focus group study among men who have sex with men emphasized that encrypted messages, discreet notifications, and neutral app design are crucial to avoid accidental disclosure of HIV-related content [44].

HIV testing is the first step in reaching those at increased risk of HIV acquisition. Our study found that 63.7% of respondents rarely undergo HIV testing, highlighting the need for frequent screening. This is especially critical in Pakistan, where documented HIV outbreaks have been linked to gaps in testing and prevention among key populations [14]. mHealth could play a vital role in assessing risks and reminding and potentially motivating the marginalized community to be tested regularly through interactive quizzes and tailored push notifications [45,46]. A randomized control trial in Vietnam showed that mHealth improved knowledge and PrEP adherence through interactive quizzes and tailored push notifications [47]. As such, innovative technology can accelerate engagement in HIV prevention strategies and facilitate the PrEP uptake and HIV self-testing, especially in areas where these services are underused [48]. Our results highlight that there is considerable interest in specific mHealth strategies, such as receiving information related to HIV prevention, e-consultation, medication reminders, and mental health information. Given the lack of public dialogue and stigma, it is not surprising that the Pakistani marginalized population, like their counterparts [49,50], particularly China [51], Vietnam [52], and Malaysia [27], expressed interest. Our study reported low to moderate HIV knowledge, and most of the participants were unaware of their HIV status and STIs, which is confirmed by another study that reported a lack of awareness and low literacy rate among people living with HIV [53], highlighting the need for effective interventions to boost awareness and education. Several studies have reported that mHealth interventions can be helpful in enhancing HIV awareness and education, such as studies from India, Nepal, and Malaysia, demonstrated greater improvement in HIV knowledge awareness, HIV testing, PrEP uptake, and behavioral outcomes among marginalized communities [18,54,55]. Similarly, a cohort study in Indonesia showed that a mobile app increased HIV knowledge from 20% to 60% among men who have sex with men, from 22% to 57% among transgender individuals, and from 49% to 74% among people who inject drugs. Additionally, HIV testing uptake rose by 31% among men who have sex with men, 49% among transgender individuals, and 26% among people who inject drugs [50]. Our study found high feasibility for a clinic-integrated mHealth app that aligns with a systematic review that found that mHealth was both feasible and acceptable in reducing HIV incidence and enhancing care engagement in gay, bisexual, and other men who have sex with men communities [56]. 60.5% of the respondents reported that they had used clinic-integrated mHealth, and some of them had experienced using the smartphone for searching health-related information, which reflects the digital revolution that is especially explosive in Asia, and the fast-paced growth of technology and use within the community [27]. However, it is crucial to contextualize these findings regarding people who inject drugs, as our sample was recruited from rehabilitation centers. Among people who inject drugs in rehabilitation and treatment programs, mHealth interventions show high feasibility and willingness, with around 97% owning mobile phones and strong engagement in text-based HIV prevention and adherence support [57]. In contrast, people who inject drugs outside treatment face major barriers, including low phone ownership, unstable housing, device loss or sale, and limited literacy, reducing mHealth uptake [58]. For example, in Tanzania, only 32% of people who inject drugs owned phones, and just 6.4% had smartphones [59]. Evidence from Malaysia similarly shows higher mobile phone and retention among people who inject drugs in harm reduction programs compared to those outside care [60]. These findings indicate that mHealth works well for stabilized people who inject drugs in treatment but requires tailored strategies to reach broader community populations. Rapid advancements in mobile technology and app development are creating new opportunities to incorporate mHealth into existing HIV prevention services in the region. A mHealth-based app could serve as an additional tool to assist marginalized communities with HIV prevention or care needs between clinic visits, guiding them to needed services, potentially enhancing clinical care, and supporting them through screening and recommendations, and providing different modes of accessing information and prevention commodities. However, the high willingness to use reported in our study may not always translate into sustained use. Studies showed that real-world factors such as the affordability of mobile data, variable digital literacy, especially among marginalized populations, strongly impact users’ ability to navigate and benefit from these apps, with digital health literacy often requiring skills beyond general health literacy [61,62]. Mistrust of technology-based health services, rooted in historical health care disparities and concerns about privacy, further reduces long-term acceptability and use [63]. Addressing these barriers through community health worker support, culturally sensitive design, and policies promoting digital literacy and affordable connectivity is critical to translating initial interest into sustained mHealth app use.

### Limitations

Despite valuable insights, this study has several limitations. First, the sample size was limited to 210 participants due to resource constraints and challenges in accessing the hard-to-reach population. A larger, more diverse sample, especially including men who have sex with men and people who inject drugs from underdeveloped areas, could improve generalizability. Moreover, interpretation of the regression analysis should be approached with caution, as several predictors, including students, HIV knowledge, and prior mHealth experience, showed wide confidence intervals likely due to small subgroup sizes. Second, key populations like sex workers and prisoners were excluded due to legal and ethical constraints, notably the criminalization of sex work under Islamic and national laws. Third, reliance on self-reported data for HIV or STI status, sexual behavior, and drug use may introduce social desirability bias, especially given the criminalization of same-sex behavior and drug abuse under Section 377 [16] and the Narcotic Control Act [64]. While self-administered, anonymous questionnaires helped reduce this bias, future studies could ensure privacy using branching logic and adaptive questioning. Fourth, because RDS weights were not applied, estimates for men who have sex with men and transgender participants reflect sample characteristics rather than population-level estimates. This may limit generalizability and affect direct comparability with the convenience sample of people who inject drugs. Furthermore, recruiting people who inject drugs via convenience sampling from rehabilitation centers may overestimate mHealth feasibility compared to the broader, nontreated populations like people who inject drugs, who likely face greater structural and digital access barriers. Findings should therefore be interpreted with these sampling differences in mind. Finally, while participants expressed a high willingness to use mHealth, stated interest may not always translate to actual usage. Future implementation studies are needed to assess real-world uptake. Although this study focused on HIV-negative individuals, mHealth also shows potential for people living with HIV, particularly in supporting ART adherence and care management [6,26,65]. Future qualitative studies involving both people living with HIV and health care providers are recommended.

### Practical Implications

#### Overview

This study offers important implications for public health programs and digital health policy in Pakistan. High smartphone ownership (76.7%) and internet access (54.8%) among the key population support the early development of the pilot mHealth intervention, with potential funding support from international donors. However, high willingness to use may not always lead to actual use, and long-term engagement may be affected by mobile data costs, varying digital literacy, and mistrust of technology-based health services [61,62]. Digital platforms offer scalable, resilient health care delivery, crucial during disruptions like those faced by HIV-positive individuals during the COVID-19 pandemic [66,67].

#### Short-Term Goals

A pilot study should be implemented to test the actual feasibility, usability, and retention in use of mHealth interventions. Given widespread digital engagement, tailored interventions such as apps or SMS text messaging–based systems with multilingual options (eg, Urdu and Punjabi) and gamified education can serve as cost-effective, discreet platforms for HIV education, medication reminders, e-consultation, and mental health resources. Considering the high rate of depressive symptoms (61.4%) and injectable drug use (58.1%), Ahmed et al [13] measured health-related quality of life among people living with HIV and also reported that 63.1% of participants were highly depressed or anxious. Apps should also ensure anonymity, encrypted interfaces, and offline accessibility, especially in a legally restrictive environment where same-sex behavior and drug use are criminalized. Low rates of HIV testing and limited knowledge (16.9%) highlight the need for mHealth tools to promote self-testing, PrEP education, and risk assessment. Push notifications and app-based tools may encourage testing and PrEP uptake, especially among high-risk groups.

#### Long-Term Goals

After the pilot stage and successful implementation of mHealth among the key population, the focus must shift toward integrating people living with HIV and other correlated programs, such as tuberculosis. This requires training health care providers to integrate these tools into ART centers and national prevention strategies. Retention in care remains a major challenge in Pakistan, especially among mobile communities who are at high risk of being lost to follow-up [68]. The app should be connected with the national database, and services like ART medication delivery through an anonymous way should be incorporated to facilitate these groups and engage them in care. To address structural barriers like violence (41.4%) and police detention (61.6%), mHealth interventions should be supported by community-based organizations that can offer legal and economic assistance. Such an app could incorporate a one-touch emergency support button connecting users to a trusted CBO focal person, a brief and low-literacy legal rights guide in Urdu, a directory of verified safe spaces such as partner clinics or drop-in centers, and secure in-app messaging for confidential safety or rights-related advice [69,70].

### Conclusions

This study aimed to assess the feasibility, willingness, and preferences for mHealth interventions among men who have sex with men, transgender individuals, and people who inject drugs in Pakistan. Our findings demonstrate high acceptability and readiness to engage with a future mHealth solution tailored to their needs. Such platforms, yet to be developed, could overcome key barriers, including stigma, low HIV knowledge, and irregular testing, by offering discreet, accessible, and user-centered services. These should include features like HIV education, medication reminders, e-consultation, and mental health support. Policymakers, in collaboration with CBOs, should develop encrypted, culturally sensitive platforms with e-consultation and mental health support aimed at enhancing reach and mitigating HIV risk. Future research, including qualitative studies, is needed to optimize app design for these populations and people living with HIV.

## Supplementary material

10.2196/81111 Multimedia Appendix 1 Sample questionnaire.

10.2196/81111 Checklist 1 STROBE checklist.

10.2196/81111 Checklist 2 SAGER checklist.

## Abbreviations

ART: antiretroviral therapy
CBO: community-based organization
mHealth: mobile health
NGO: nongovernmental organization
OR: odds ratio
PrEP: pre-exposure prophylaxis
RDS: respondent-driven sampling
SAGER: Sex and Gender Equity Research
SPSS: Statistical Package for the Social Sciences
STI: sexually transmitted infection
STROBE: Strengthening the Reporting of Observational Studies in Epidemiology
UNDP: United Nations Development Programme
WHO: World Health Organization

## References

[1] Gautam K, Paudel K, Ahmed A (2024). High interest in the use of mHealth platform for HIV prevention among men who have sex with men in Nepal. J Community Health.

[2] (2023). Global HIV & AIDS statistics fact sheet 2023. Joint United Nations Programme on HIV/AIDS.

[3] (2023). Executive summary—the path that ends AIDS: UNAIDS global AIDS update 2023. Joint United Nations Programme on HIV/AIDS.

[4] (2022). Dangerous inequalities: World AIDS Day report 2022. Joint United Nations Programme on HIV/AIDS.

[5] Lyons CE, Schwartz SR, Murray SM (2020). The role of sex work laws and stigmas in increasing HIV risks among sex workers. Nat Commun.

[6] Krishnan A, Weikum D, Cravero C, Kamarulzaman A, Altice FL (2021). Assessing mobile technology use and mHealth acceptance among HIV-positive men who have sex with men and transgender women in Malaysia. PLoS ONE.

[7] Chandler R, Guillaume D, Francis S (2023). “I care about sex, I care about my health”: a mixed-methods pre-test of a HIV prevention mobile health app for Black women in the southern United States. PLoS One.

[8] Huang H, Xu Z, Ge Q (2023). The impact of customized short message service on high-risk behaviors among MSM in China, a randomized controlled trial study. AIDS Behav.

[9] (2025). National AIDS control programme (NACP). Common Management Unit.

[10] Banik S, Khan S, Jami H, Sivasubramanian M, Dhakal M, Wilson E (2023). Social Determinants of Sexual Health Among Sexual and Gender Diverse People in South Asia: Lessons Learned from India.

[11] Bhatti MW (2025). Global fund cuts aid to Pakistan despite rising HIV, TB, malaria. Geo News.

[12] (2022). National AIDS control programme of Pakistan. Ministry of National Health Services Regulation and Coordination, Government of Pakistan.

[13] Ahmed A, Saqlain M, Bashir N (2021). Health-related quality of life and its predictors among adults living with HIV/AIDS and receiving antiretroviral therapy in Pakistan. Qual Life Res.

[14] Ahmed A, Hashmi FK, Khan GM (2019). HIV outbreaks in Pakistan. Lancet HIV.

[15] Raza HA, Raja MHR, Khakwani MM, Jamil B (2024). Pakistan’s HIV high-risk populations: critical appraisal of failure to curtail spread beyond key populations. IJID Reg.

[16] Khan FA, Wadle SS (2014). South Asia in the World: An Introduction.

[17] Kemp S (2025). Digital 2025: Pakistan. DataReportal.

[18] Shrestha R, Altice FL, Khati A (2023). Clinic-integrated smartphone app (JomPrEP) to improve uptake of HIV testing and pre-exposure prophylaxis among men who have sex with men in Malaysia: mixed methods evaluation of usability and acceptability. JMIR Mhealth Uhealth.

[19] Hayat MO (2024). Sehat Dost: digital solutions to HIV-related discrimination. United Nations Development Programme.

[20] Egbuchulem KI (2023). The basics of sample size estimation: an editor’s view. Ann Ib Postgrad Med.

[21] Althubaiti A (2022). Sample size determination: a practical guide for health researchers. J Gen Fam Med.

[22] Naing L, Nordin RB, Abdul Rahman H, Naing YT (2022). Sample size calculation for prevalence studies using Scalex and ScalaR calculators. BMC Med Res Methodol.

[23] (2011). Global health sector strategy on HIV/AIDS 2011–2015. https://iris.who.int/server/api/core/bitstreams/0918c708-f163-4886-8adc-461d67a72fb2/content.

[24] (2016). Differentiated service delivery for key populations: men who have sex with men, sex workers, transgender people, people who inject drugs and prisoners and other people living in closed settings: a background review. Differentiated Service Delivery.

[25] (2016). Consolidated guidelines on HIV prevention, diagnosis, treatment and care for key populations—2016 update. https://iris.who.int/server/api/core/bitstreams/918a4a9a-5116-49d7-9074-e7bcf295d0b6/content.

[26] Irfan A (2023). Finding the gaps in stories of sex workers in Pakistan. New Line Magazine.

[27] Shrestha R, Maviglia F, Altice FL (2022). Mobile health technology use and the acceptability of an mHealth platform for HIV prevention among men who have sex with men in Malaysia: cross-sectional respondent-driven sampling survey. J Med Internet Res.

[28] Heidari S, Babor TF, De Castro P, Tort S, Curno M (2024). Publisher correction: Sex and Gender Equity in Research: rationale for the SAGER guidelines and recommended use. Res Integr Peer Rev.

[29] Shrestha R, Altice FL, DiDomizio E, Sibilio B, Ranjit YS, Copenhaver MM (2020). Feasibility and acceptability of an mHealth-based approach as an HIV prevention strategy among people who use drugs on pre-exposure prophylaxis. Patient Prefer Adherence.

[30] Rawat S, Wilkerson JM, Lawler SM (2018). Recommendations for the development of a mobile HIV prevention intervention for men who have sex with men and Hijras in Mumbai: qualitative study. JMIR Public Health Surveill.

[31] Reza MM, Rana AM, Azim T (2020). Changes in condom use among males who have sex with males (MSM): measuring the effect of HIV prevention programme in Dhaka city. PLoS One.

[32] Harjana NPA, Lubis DS, Fauk NK, Gesesew HA, Ward PR, Januraga PP (2024). Health information-seeking behaviour, intimate partner violence, and sexual risk-taking among MSM in Bali, Indonesia. J Men’s Health.

[33] Abdul Manaf R, Dickson N, Lovell S, Ibrahim F (2019). Consistent condom use and its predictors among female sexual partners of people who inject drugs in Klang Valley, Malaysia. BMC Public Health.

[34] Fauk NK, Kustanti CY, Liana DS, Indriyawati N, Crutzen R, Mwanri L (2018). Perceptions of determinants of condom use behaviors among male clients of female sex workers in Indonesia: a qualitative inquiry. Am J Mens Health.

[35] Choi EPH, Wu C, Choi KWY (2024). Ehealth interactive intervention in promoting safer sex among men who have sex with men. NPJ Digit Med.

[36] Isaacs N, Ntinga X, Keetsi T (2024). Are mHealth interventions effective in improving the uptake of sexual and reproductive health services among adolescents? A scoping review. Int J Environ Res Public Health.

[37] Hart TA, Noor SW, Berlin GW (2023). Pre-exposure prophylaxis and bacterial sexually transmitted infections (STIs) among gay and bisexual men. Sex Transm Infect.

[38] Yan X, Jia Z, Zhang B (2022). Evaluating the risk compensation of HIV/AIDS prevention measures. Lancet Infect Dis.

[39] Baek YJ, Lee Y, Lee JA (2025). Sexual risk compensation and retention in PrEP care in Korea: an HIV PrEP demonstration study. J Korean Med Sci.

[40] To KW, Lee SS (2018). HIV pre-exposure prophylaxis in South East Asia: a focused review on present situation. Int J Infect Dis.

[41] Abboud S, Veldhuis C, Ballout S, Nadeem F, Nyhan K, Hughes T (2022). Sexual and gender minority health in the Middle East and North Africa region: a scoping review. Int J Nurs Stud Adv.

[42] Ameli V, Haberer J, Sabin L (2021). Tailored mHealth intervention for improving treatment adherence for people living with HIV in Iran (HamRaah): protocol for a feasibility study and randomised pilot trial with a nested realist evaluation. BMJ Open.

[43] Shrestha R, Fisher C, Wickersham JA (2021). Privacy and confidentiality concerns related to the use of mHealth apps for HIV prevention efforts among Malaysian men who have sex with men: cross-sectional survey study. JMIR Form Res.

[44] Khati A, Wickersham JA, Rosen AO (2022). Ethical issues in the use of smartphone apps for HIV prevention in Malaysia: focus group study with men who have sex with men. JMIR Form Res.

[45] Sanabria G, Scherr T, Garofalo R (2021). Usability evaluation of the mLab app for improving home HIV testing behaviors in youth at risk of HIV infection. AIDS Educ Prev.

[46] Biello KB, Horvitz C, Mullin S (2021). HIV self-testing and STI self-collection via mobile apps: experiences from two pilot randomized controlled trials of young men who have sex with men. Mhealth.

[47] Tran BX, Bui TM, Do AL (2023). Efficacy of a mobile phone–based intervention on health behaviors and HIV/AIDS treatment management: randomized controlled trial. J Med Internet Res.

[48] Biello KB, Hill-Rorie J, Valente PK (2021). Development and evaluation of a mobile app designed to increase HIV testing and pre-exposure prophylaxis use among young men who have sex with men in the United States: open pilot trial. J Med Internet Res.

[49] Lu X, Gao P, Wang X (2021). User preferences for an mHealth approach to support HIV self-testing and linkage to HIV prevention or care services for MSM in China. AIDS Educ Prev.

[50] Garg PR, Uppal L, Mehra S, Mehra D (2020). Mobile health app for self-learning on HIV prevention knowledge and services among a young Indonesian key population: cohort study. JMIR Mhealth Uhealth.

[51] Marley G, Fu G, Zhang Y (2021). Willingness of Chinese men who have sex with men to use smartphone-based electronic readers for HIV self-testing: web-based cross-sectional study. J Med Internet Res.

[52] Trang K, Le LX, Brown CA (2022). Feasibility, acceptability, and design of a mobile ecological momentary assessment for high-risk men who have sex with men in Hanoi, Vietnam: qualitative study. JMIR Form Res.

[53] Aizaz M, Abbas FA, Abbas A, Tabassum S, Obeagu EI (2023). Alarming rise in HIV cases in Pakistan: challenges and future recommendations at hand. Health Sci Rep.

[54] Ayer R, Poudel KC, Kikuchi K, Ghimire M, Shibanuma A, Jimba M (2021). Nurse-led mobile phone voice call reminder and on-time antiretroviral pills pick-up in Nepal: a randomized controlled trial. AIDS Behav.

[55] Patel VV, Rawat S, Dange A, Lelutiu-Weinberger C, Golub SA (2020). An internet-based, peer-delivered messaging intervention for HIV testing and condom use among men who have sex with men in India (CHALO!): pilot randomized comparative trial. JMIR Public Health Surveill.

[56] Nelson KM, Perry NS, Horvath KJ, Smith LR (2020). A systematic review of mHealth interventions for HIV prevention and treatment among gay, bisexual, and other men who have sex with men. Transl Behav Med.

[57] Chandrasekaran S, Kyaw NTT, Harries AD (2017). Enrolment and retention of people who inject drugs in the Needle & Syringe Exchange Programme in Malaysia. Public Health Action.

[58] Pang J, Danaee M, Balasingam Kasinather V, Des Jarlais D, Kamarulzaman A, Mohd Salleh NA (2025). Current drug use patterns and HIV and HCV prevalence among people who inject drugs in suburban areas of Malaysia. J Int AIDS Soc.

[59] Munishi C, Ndumwa HP, Massawe JE (2023). Acceptability of using mobile Health (mHealth) as an intervention tool for people with drug use disorders in Tanga, Tanzania. PLOS Digit Health.

[60] Kumar D, Hemmige V, Kallen MA, Giordano TP, Arya M (2019). Mobile phones may not bridge the digital divide: a look at mobile phone literacy in an underserved patient population. Cureus.

[61] Smith B, Magnani JW (2019). New technologies, new disparities: the intersection of electronic health and digital health literacy. Int J Cardiol.

[62] Kruse C, Betancourt J, Ortiz S, Valdes Luna SM, Bamrah IK, Segovia N (2019). Barriers to the use of mobile health in improving health outcomes in developing countries: systematic review. J Med Internet Res.

[63] Giebel GD, Speckemeier C, Abels C (2023). Problems and barriers related to the use of digital health applications: scoping review. J Med Internet Res.

[64] (1997). Control of Narcotic Substances Act, 1997 (Act no.XXV of 1997). https://www.unodc.org/cld/uploads/res/document/pak/control-of-narcotic-substances-act-xxv_html/CONTROL_OF_NARCOTIC_SUBSTANCES_ACT_pakistan.pdf.

[65] Escobar-Viera C, Zhou Z, Morano JP (2020). The Florida Mobile Health Adherence Project for people living with HIV (FL-mAPP): longitudinal assessment of feasibility, acceptability, and clinical outcomes. JMIR Mhealth Uhealth.

[66] Raza A, Ullah I, Tahir MJ, Jabbar A, Ahmed A (2022). Coronavirus disease 2019 (COVID-19) is a healthcare dilemma for human immunodeficiency virus (HIV)-positive individuals in Pakistan. Infect Control Hosp Epidemiol.

[67] Shah SA, Altaf A, Vermund SH (2021). Challenges of antiretroviral treatment for human immunodeficiency virus infection in Pakistan. J Pak Med Assoc.

[68] Ahmed A, Dujaili JA, Jabeen M (2022). Barriers and enablers for adherence to antiretroviral therapy among people living with HIV/AIDS in the era of COVID-19: a qualitative study from Pakistan. Front Pharmacol.

[69] Terlikbayeva A, Primbetova S, Gatanaga OS (2025). UMAI-WINGS: Evaluating the effectiveness of implementing mHealth intimate partner violence prevention intervention in reducing intimate partner violence among women from key affected populations in Kazakhstan using a community-based approach. Behav Sci (Basel).

[70] Lalla-Edward ST, Mashabane N, Stewart-Isherwood L (2022). Implementation of an mHealth app to promote engagement during HIV care and viral load suppression in Johannesburg, South Africa (iThemba Life): pilot technical feasibility and acceptability study. JMIR Form Res.

