# Supplementary material for: Feasibility and Preferences to Adopt mHealth-Based Interventions for HIV Prevention Among High-Risk Groups: Cross-Sectional Study
Source: JMIR Hum Factors. 2026 Mar 12;13:e81111. doi: 10.2196/81111 (PMC12981546; doi:10.2196/81111)
Supplement: Multimedia Appendix 1 [file humanfactors-v13-e81111-s001.docx]

**FEASIBILITY AND PREFERENCES TO ADOPT MOBILE HEALTH-BASED INTERVENTIONS FOR HIV PREVENTION: A CROSS-SECTIONAL STUDY AMONG HIGH-RISK GROUPS IN PAKISTAN**

**Introduction**

You are invited to participate in a research study. Before you decide, you need to understand why the research is being done and what it will involve. Please read the following information carefully and feel free to ask any questions you may have.

**تعارف**

آپ کو ایک تحقیقی مطالعہ میں شرکت کے لیے مدعو کیا جاتا ہے فیصلہ کرنے سے پہلے، آپ کو یہ سمجھنے کی ضرورت ہے کہ تحقیق کیوں کی جا رہی ہے اور اس میں کیا شامل ہوگا براہ کرم درج ذیل معلومات کو غور سے پڑھیں اور بلا جھجھک اپنے کوئی سوال پوچھیں

**Purpose of the Study**

This study aims to understand how acceptable and feasible it is to use a mobile health (mHealth) application linked to clinics for HIV prevention services. We are especially interested in hearing from people who identify as men who have sex with men (MSM), transgender individuals, and people who inject drugs (PWID). Your input will help us design digital tools that are more responsive to community needs.

**اس مطالعے کا مقصد**

اس مطالعہ کا مقصد یہ سمجھنا ہے کہ ایچ آئی وی سے بچاؤ کی خدمات کے لیے کلینک سے منسلک موبائل ہیلتھ (mHealth) ایپلیکیشن کا استعمال کتنا قابل قبول اور قابل عمل ہے۔ ہم خاص طور پر ان لوگوں سے سننے میں دلچسپی رکھتے ہیں جو مردوں کے ساتھ جنسی تعلق رکھنے والے مردوں (MSM)، ٹرانس جینڈر افراد، اور منشیات (PWID) کے انجیکشن لگانے والے افراد کے طور پر شناخت کرتے ہیں۔ آپ کا ان پٹ ہمیں ایسے ڈیجیٹل ٹولز ڈیزائن کرنے میں مدد کرے گا جو کمیونٹی کی ضروریات کے لیے زیادہ ذمہ دار ہوں۔

**What Will You Be Asked to Do?**

If you agree to participate, you will be asked to:

- Complete a short questionnaire (about 15–20 minutes) about your HIV prevention needs, mobile phone use, and interest in mHealth.

**آپ سے کیا کرنے کو کہا جائے گا؟**

اگر آپ حصہ لینے پر راضی ہیں تو آپ سے کہا جائے گا:

- ایچ آئی وی سے بچاؤ کی اپنی ضروریات، موبائل فون کے استعمال، اور ایم ہیلتھ میں دلچسپی کے بارے میں ایک مختصر سوالنامہ (تقریباً ۱۵-۲۰ منٹ) مکمل کریں

**Voluntary Participation**

Participation in this study is entirely voluntary. You may decline to participate or withdraw from the study at any time without penalty or loss of benefits to which you are otherwise entitled

**رضاکارانہ شرکت**

اس مطالعہ میں شرکت مکمل طور پر رضاکارانہ ہے آپ کسی بھی وقت اس مطالعہ میں حصہ لینے یا اس سے دستبردار ہونے سے انکار کر سکتے ہیں بغیر جرمانے یا فوائد کے نقصان کے جن کے آپ دوسری صورت میں حقدار ہیں

**Risks and Benefits**

There are minimal risks associated with this study. You may feel uncomfortable answering some questions about HIV or mobile phone use. You are free to skip any question or stop at any time. There is no direct benefit to you, but your responses will help improve HIV prevention tools for your community.

**خطرات اور فوائد**

اس مطالعے سے وابستہ کم سے کم خطرات ہیں آپ ایچ آئی وی یا موبائل فون کے استعمال کے بارے میں کچھ سوالات کے جواب دینے میں بے چینی محسوس کر سکتے ہیں آپ کسی بھی سوال کو چھوڑنے یا کسی بھی وقت رکنے کے لیے آزاد ہیں آپ کو براہ راست کوئی فائدہ نہیں ہے، لیکن آپ کے جوابات آپ کی کمیونٹی کے لیے ایچ آئی وی سے بچاؤ کے آلات کو بہتر بنانے میں مدد کریں گے

**Confidentiality**

All the information you provide will be kept strictly confidential. Your name or any identifying information will not appear in any report or publication. Data will be stored securely and used only for research purposes.

**رازداری**

آپ کی فراہم کردہ تمام معلومات کو سختی سے خفیہ رکھا جائے گا آپ کا نام یا کوئی شناختی معلومات کسی رپورٹ یا اشاعت میں ظاہر نہیں ہوگی ڈیٹا کو محفوظ طریقے سے ذخیرہ کیا جائے گا اور صرف تحقیقی مقاصد کے لیے استعمال کیا جائے گا

| **حصہ ۱: آبادیاتی حیثیت** | **Section 1: Demographic Status** |
| --- | --- |
| 1. عمر____________________ | **1. Age:** ______________________ |
| 1. **کلیدی آبادیاں** 2. مرد جو مردوں کے ساتھ جنسی تعلق رکھتے ہیں 3. ٹرانس جینڈر افراد 4. منشیات بذریعہ انجیکشن استعمال کرنے والے افراد | **2. Key populations**   1. Men who have sex with men 2. Transgender individuals 3. People who inject drugs |
| 1. **جنسی رجحان** 2. مخالف جنس پرست 3. ہم جنس پرست 4. دو جنس پرست 5. دیگر (براہ کرم وضاحت کریں _____________________ | **3. Sexual Orientation**   1. Heterosexual 2. Homosexual 3. Bisexual 4. Other (Please Specify) |
| 1. **تعلیمی سطح** 2. ہائی اسکول سے کم 3. ہائی اسکول 4. کالج/ایسوسی ایٹ ڈگری 5. بیچلر ڈگری 6. گریجویٹ/پروفیشنل ڈگری | **4. Education Level**   1. Less than high school 2. High school 3. College/associate’s degree 4. Bachelor’s degree 5. Graduate/Professional degree |
| 1. **ملازمت کی حیثیت**    1. کل وقتی ملازم    2. جز وقتی ملازم    3. بے روزگار    4. طالب علم | **5. Employment Status**   - 1. Employed full time   2. Employed part time   3. Unemployed   4. Student |
| 1. **قومیت/مذہب** 2. مسلمان 3. غیر مسلم | **6. Ethnicity/Race**   1. Muslim 2. Non-Muslim |
| **حصہ ۲: ایچ آئی وی آگاہی اور علم** | **Section 2: HIV Awareness and Knowledge** |
| 1. **کیا آپ ایچ آئی وی اور اس کے پھیلاؤ کے طریقوں سے واقف** ہیں؟ 2. ہاں 3. نہیں | **7. Are you aware of HIV and its transmission methods?**   1. Yes 2. No |
| 1. **۱ (بہت کم) سے ۵ (بہت زیادہ) کے پیمانے پر، آپ ایچ آئی وی کے بارے میں اپنے علم کو کیا درجہ دیں** گے؟ 2. بہت کم 3. کم 4. معتدل 5. زیادہ 6. بہت زیادہ | **8. On scale from 1 (very low) to 5 (very high), how would you rate your knowledge about HIV?**   1. Very Low 2. Low 3. Moderate 4. high 5. Very high |
| **حصہ ۳: موبائل ایپ اور ٹیکنالوجی کا استعمال** | **Section 3: Mobile App and Technology Usage** |
| 1. **کیا آپ کے پاس اسمارٹ فون ہے؟**  - **ہاں** - **نہیں** | **9. Do you own a smartphone?**   - Yes - No |
| 1. **کیا آپ کو روزانہ انٹرنیٹ تک رسائی حاصل ہے؟**  - **ہاں** - **نہیں** | **10. Have you had daily access to the internet?**   - Yes - No |
| 1. **آپ انٹرنیٹ پر کتنا وقت صرف کرتے ہیں؟ (گھنٹے/دن)** ____________________________ | **11. Have much time do you spend on the internet?** (Hours/day) _____________________________ |
| 1. **انٹرنیٹ پر عام طور پر کون سی سرگرمیاں کی جاتی ہیں؟**  - آن لائن سماجی نیٹ ورکنگ - ای میل بھیجنا یا وصول کرنا - جی ایس این ایپس/ویب سائٹس - صحت سے متعلق ایپس کا استعمال | **12. What activities are usually done on the Internet?**   - Online Social Networking - Send or receive emails - GSN apps/Websites - Use health-related apps |
| 1. **آپ اپنے اسمارٹ فون پر موبائل ایپس کتنی کثرت سے استعمال کرتے ہیں؟**  - روزانہ - ہفتے میں کئی بار - ہفتے میں ایک بار - کبھی کبھار - کبھی نہیں | **13. How frequently do you use mobile apps on your smartphone?**   - Daily - Several times a week - Once a week - Rarely - Never |
| 1. **کیا آپ نے کبھی صحت کی دیکھ بھال یا ایچ آئی وی سے بچاؤ سے متعلق کوئی موبائل ایپ استعمال کی ہے؟**  - **ہاں** - **نہیں** | **14. Have you ever used a mobile app related to healthcare or HIV prevention?**   - Yes - No |
| 1. **اگر مذکورہ سوال (یعنی سوال 14) کا جواب ہاں میں ہے، تو آپ صحت کی دیکھ بھال یا ایچ آئی وی سے بچاؤ کی موبائل ایپس استعمال کرنے کے اپنے تجربے سے کس حد تک مطمئن ہیں؟**    1. بہت غیر مطمئن    2. غیر مطمئن    3. غیر جانبدار    4. مطمئن    5. بہت مطمئن | **15. If yes for the above question 12, how satisfied are you with your experience using healthcare or HIV prevention mobile apps?**   1. Very Dissatisfied 2. Dissatisfied 3. Neutral 4. Satisfied 5. Very Satisfied |
| 1. **کیا آپ ایچ آئی وی سے بچاؤ یا دیکھ بھال کے لیے کلینک سے مربوط موبائل ایپ استعمال کرنے پر رضامند ہوں گے؟**  - **ہاں** - **نہیں** | **16. Would you be willing to use a clinic-integrated mobile app for HIV prevention or care?**   - Yes - No |
| 1. **کیا آپ ادویات کی یاد دہانیاں وصول کرنے میں دلچسپی رکھتے ہیں؟ اگر ہاں، تو آپ کی ترجیحی تعداد کیا ہے؟**  - روزانہ - ہفتہ وار - ماہانہ - کبھی نہیں | **17. Are you interested in receiving medication reminders? If yes, what is your preferred frequency?**   - Daily - Weekly - Monthly - Never |
| 1. **یاد دہانیاں وصول کرنے کا آپ کا ترجیحی طریقہ کار کیا ہے؟**  - فون کالز - ٹیکسٹ میسج (پیغام) - ایپ نوٹیفکیشن (اطلاع) - ویب سائٹس | **18. What is your preferred mechanism of getting reminders?**   - Phone calls - Text message - App notification - Websites |
| 1. **آپ کتنی کثرت سے ایچ آئی وی سے بچاؤ کی معلومات حاصل کرنا چاہیں گے؟**  - روزانہ - ہفتہ وار - ماہانہ - کبھی نہیں | **19. How frequently would you like to receive HIV prevention information?**   - Daily - Weekly - Monthly - Never |
| 1. **ایچ آئی وی سے بچاؤ کی معلومات حاصل کرنے کا آپ کا ترجیحی طریقہ کار کیا ہے؟**  - فون کالز - ٹیکسٹ میسجز (پیغامات) - ایپ - ویب سائٹ | **20. What is your preferred mechanism of receiving HIV prevention information?**   - Phone calls - Text messages - App - Website |
| 1. **کیا آپ ڈاکٹر سے ای-مشاورت (برقی مشاورت) حاصل کرنا چاہیں گے؟** | **21. Would you like to get an e-consultation with the doctor?**   - Yes - No |
| 1. **ای-مشاورت حاصل کرنے کا آپ کا ترجیحی طریقہ کار کیا ہے؟**  - فون کالز - ٹیکسٹ میسجز - ایپ - ویب سائٹ | **22. What is your preferred mechanism of getting e-consultation?**   - Phone calls - Text messages - App - Website |
| 1. **کیا آپ ذہنی صحت سے متعلق معلومات حاصل کرنا چاہیں گے؟**  - **ہاں** - **نہیں** | **23. Would you like to receive information on mental health?**   - Yes - No |
| 1. **ذہنی صحت سے متعلق معلومات حاصل کرنے کا آپ کا ترجیحی طریقہ کار کیا ہے؟**  - فون کالز - ٹیکسٹ میسجز - ایپ - ویب سائٹ | **24. What is your preferred mechanism of receiving information on mental health?**   - Phone calls - Text messages - App - Website |
| 1. **کیا آپ ذہنی صحت کے لیے مجازی (ورچوئل) سپورٹ گروپ میں شامل ہونا چاہیں گے؟**  - **ہاں** - **نہیں** | **25. Would you like to engage yourself in a virtual support group for mental health?**   - Yes - No |
| **حصہ 4: ایچ آئی وی سے بچاؤ اور دیکھ بھال** | **Section 4: HIV Prevention and Care**: |
| 1. **کیا آپ نے کبھی ایچ آئی وی کا ٹیسٹ کروایا ہے؟**  - **ہاں** - **نہیں** | **26. Have you ever been tested for HIV?**   - Yes - No |
| 1. **اگر ہاں، تو آپ کتنی بار ایچ آئی وی کا ٹیسٹ کرواتے ہیں؟**    1. باقاعدگی سے) سالانہ(    2. کبھی کبھار) ہر چند سال بعد(    3. بہت کم | **27. If yes for question 12, how often do you get tested for HIV?**   - Regularly (e.g., annually) - Occasionally (e.g., every few years) - Rarely |
| 1. **آپ کی ایچ آئی وی کی حیثیت کیا ہے؟**    1. مثبت    2. منفی    3. معلوم نہیں | **28. What is your HIV status?**   1. Positive 2. Negative 3. Don’t know |
| 1. **کیا آپ فی الحال ایچ آئی وی سے بچاؤ یا دیکھ بھال کی کوئی خدمات حاصل کر رہے ہیں؟**  - **ہاں** - **نہیں** | **29. Are you currently receiving any form of HIV prevention or care services?**   - Yes - No |
| 1. **اگر ہاں، تو براہ کرم ان خدمات کی اقسام بیان کریں** _________________________________ | **30. If yes, please specify the types of HIV prevention or care services you are receiving.**  _________________________________ |
| 1. **کیا آپ کو کبھی جنسی طور پر منتقل ہونے والی بیماری کی تشخیص ہوئی ہے؟**  - **ہاں** - **نہیں** - **پتہ نہیں** | **31. Have you ever been diagnosed with a sexually transmitted infection (STI)?**   - Yes - No - Don’t know |
| 1. **کیا آپ نے کبھی قبل از وقوع احتیاطی تدابیر (PrEP) استعمال کی ہیں؟**  - **ہاں** - **نہیں** | **32. Have you ever used pre-exposure prophylaxis (PrEP)?**   - Yes - No |
| 1. **کیا آپ نے کبھی بعد از وقوع احتیاطی تدابیر (PEP) استعمال کی ہیں؟**  - **ہاں** - **نہیں** | **33. Have you ever used post-exposure prophylaxis (PEP)?**   - Yes - No |
| 1. **کیا آپ نے ماضی میں تشدد کا تجربہ کیا ہے؟**  - **ہاں** - **نہیں** | **34. Have you experienced violence in the past?**   - Yes - No |
| 1. **کیا آپ کو کبھی پولیس نے حراست میں لیا ہے؟**  - **ہاں** - **نہیں** | **35. Have you ever been detained by the police?**   - Yes - No |
| 1. **کیا آپ فی الحال افسردگی کی علامات کا تجربہ کر رہے ہیں؟**  - **ہاں** - **نہیں** | **36. Do you currently experience depressive symptoms?**   - Yes - No |
| 1. **کیا آپ نے کبھی منشیات بذریعہ انجیکشن استعمال کی ہیں؟**  - **ہاں** - **نہیں** | **37. Have you ever injected drugs?** ؟   - Yes - No |
| 1. **کیا آپ نے گزشتہ 6 مہینوں میں مقعدی جنسی عمل میں حصہ لیا ہے؟**  - **ہاں** - **نہیں** | **38. Have you engaged in anal sex in the past 6 months?** ؟   - Yes - No |
| 1. **کیا گزشتہ 6 مہینوں میں آپ کے ایک سے زیادہ جنسی ساتھی رہے ہیں؟**  - **ہاں** - **نہیں** | **39. Have you had multiple sex partners in the past 6 months?** ؟   - Yes - No |
| 1. **کیا آپ نے گزشتہ6 مہینوں میں لین دین پر مبنی جنسی عمل میں حصہ لیا ہے؟**  - **ہاں** - **نہیں** | **40. Have you engaged in transactional sex in the past 6 months?**   - Yes - No |
| 1. **کیا آپ نے گزشتہ 6 مہینوں میں جنسی تعلقات کے دوران مستقل طور پر کنڈوم استعمال کیا ہے؟**  - **ہاں** - **نہیں** | **41. Have you consistently used condoms in sexual encounters in the past 6 months?**   - Yes - No |
| **حصہ ۵: ایچ آئی وی سے بچاؤ اور دیکھ بھال کے لیے موبائل ایپس کی فزیبلٹی** | **Section 5: Feasibility of Mobile Apps for HIV Prevention and Care** |
| 1. **ایچ آئی وی سے بچاؤ اور دیکھ بھال کے لیے کلینک سے مربوط موبائل ایپ میں آپ کن خصوصیات کو سب سے اہم سمجھتے ہیں؟** 2. اپائنٹمنٹ شیڈولنگ 3. ادویات کی یاد دہانیاں 4. ایچ آئی وی ٹیسٹنگ کی معلومات 5. تعلیمی وسائل 6. صحت کی دیکھ بھال فراہم کرنے والوں کے ساتھ محفوظ پیغام رسانی 7. دیگر (براہ کرم وضاحت کریں) | **42. What features would you consider most important in a clinic-integrated mobile app for HIV prevention and care? (Select all that apply)**   1. Appointment Scheduling 2. Medication Reminders 3. HIV testing information 4. Educational Resources 5. Secure messaging with healthcare providers 6. Other (Please specify) ……………………… |
| 1. **۱ سے ۵ کے پیمانے پر، آپ کے خیال میں موبائل ایپس کو ایچ آئی وی سے بچاؤ اور دیکھ بھال کی خدمات میں ضم کرنا کتنا قابل عمل ہے؟**    1. بالکل قابل عمل نہیں    2. تھوڑا سا قابل عمل    3. معمولی طور پر قابل عمل    4. بہت قابل عمل    5. انتہائی قابل عمل | **43. On a scale 1 to 5 how feasible do you think it is to integrate mobile apps into HIV prevention and care services?**   1. Not at all feasible 2. Slightly feasible 3. Moderately feasible 4. Very feasible 5. Extremely feasible |
