# Supplementary material for: Feasibility and Preferences to Adopt mHealth-Based Interventions for HIV Prevention Among High-Risk Groups: Cross-Sectional Study
Source: JMIR Hum Factors. 2026 Mar 12;13:e81111. doi: 10.2196/81111 (PMC12981546; doi:10.2196/81111)
Supplement: Checklist 2 [file humanfactors-v13-e81111-s003.docx]

# SAGER Checklist – Sex and Gender Equity in Research

This checklist is completed in accordance with the SAGER guidelines (Heidari et al., 2016) to ensure sex and gender equity in the reporting of research.

| - **Research approaches** |
| --- |
| 1. **Are the concepts of gender and/or sex used in your research project?** |
| Yes, the research explicitly involved gender and sexual identity-based populations, including men who have sex with men (MSM), transgender (TG) individuals, and people who inject drugs (PWIDs). The study explored how mHealth tools can be tailored for HIV prevention in these groups, acknowledging their unique social and structural vulnerabilities related to gender and sexual identity. |
| 1. **If yes, have you explicitly defined the concepts of gender and/or sex? Is it clear what aspects of gender and/or sex are being examined in your study?** |
| The manuscript provided clear definitions of key populations based on internationally accepted terminology (WHO). MSM was defined behaviorally, regardless of sexual identity, while TG individuals were described as persons whose gender identity did not conform to their assigned sex at birth. The study examined how gender identity (e.g., transgender women) and sexual behavior (e.g., MSM) relate to HIV risk behaviors, mental health (depressive symptoms), legal vulnerability (police detention), and engagement with mHealth tools.  The study did not focus on biological sex as a variable but instead emphasized gender identity and sexual behavior as they pertain to public health engagement and barriers. |
| 1. **If not, do you consider this to be a significant limitation? Given existing knowledge in the relevant literature, are there plausible gender and/or sex factors that should have been considered? If you consider sex and/or gender to be highly relevant to your proposed research, the research design should reflect this** |
| This question was not applicable, as the concepts of gender and sexual identity were central to the study design, analysis, and interpretation. The research design intentionally targeted populations most at risk due to gender- and behavior-based marginalization, and these identities were central to both recruitment and analysis. |
| - **Research questions and hypotheses** |
| 1. **Does your research question(s) or hypothesis/es make reference to gender and/or sex, or relevant groups or phenomena?** |
| The central aim of the study was to assess the willingness, preferences, and feasibility of mHealth interventions for HIV prevention among key populations in Pakistan, specifically:   - Men who have sex with men (MSM) - Transgender (TG) individuals - People who inject drugs (PWIDs)   These groups were recognized as gender- and behaviorally distinct populations that faced disproportionate HIV risk due to stigma, legal criminalization, and structural inequities.  Although the research questions were not framed in a traditional hypothesis-testing format (e.g., "we hypothesize that..."), the implicit hypotheses were clearly structured around differences in:   - mHealth accessibility and preferences - HIV prevention behaviors - Mental health symptoms - Exposure to violence and detention, ...all as they relate to gender identity or marginalized sexual behaviors. |
| - **Literature review** |
| 1. **Does your literature review cite prior studies that support the existence (or lack) of significant differences between women and men, boys and girls, or males and females?** |
| Our literature review did not explicitly compare males vs. females in a binary biological sex framework, but it did cite multiple studies that supported the existence of disparities based on gender identity and sexual behavior, especially among:   - Transgender women - MSM   People who inject drugs (PWIDs)  UNAIDS and WHO data showing that transgender individuals have a 14 times higher risk, and MSM an 11 times higher risk, of acquiring HIV compared to the general adult population. Additionally, studies and meta-analyses highlighted higher prevalence of depression, substance use, and legal vulnerabilities among gender and sexual minorities.  So while the review didn’t compare "males vs. females" in the biological sense, it clearly supported gender-based disparities and contextualized the elevated HIV risk among gender-diverse and behaviorally marginalized groups. |
| 1. **Does your literature review point to the extent to which past research has taken gender or sex into account?** |
| mHealth interventions for HIV prevention were mostly studied in high-income countries, with limited data available for gender and sexual minorities in low- and middle-income countries (LMICs), including Pakistan. We specifically identify a research gap in how previous studies have addressed the feasibility and acceptability of mHealth tools among MSM, TG individuals, and PWIDs in the local context.  Additionally:   - Studies showing that gender minorities were more affected by legal stigma. - The lack of inclusive digital platforms in Pakistan that considered the needs of these populations.   This positions our study as a response to the lack of gender-sensitive digital health research in LMIC settings. |
| - **Research method** |
| 1. **Is your sample appropriate to capture gender and/or sex-based factors?** |
| Yes, the sample was explicitly designed to include individuals from key genders and behaviorally defined populations:   - MSM (men who have sex with men) - Transgender (TG) individuals - People who inject drugs (PWIDs)   These groups were selected due to their high HIV burden, their exclusion from mainstream health research, and their relevance to understanding gender-related barriers in HIV prevention. The sampling strategy, Respondent-driven sampling (RDS) and convenience sampling through CBOs, were specifically tailored to reach these marginalized and stigmatized gender/behavioral groups in Pakistan. |
| 1. **Is it possible to collect data that are disaggregated by sex and/or gender?** |
| Yes, the study collected self-reported data on gender identity, allowing for disaggregation by MSM, TG individuals, and PWIDs. While biological sex was not the focus, the gender identity categories captured in the survey are appropriate for the populations studied and align with current gender-sensitive public health approaches. |
| 1. **Are the inclusion and exclusion criteria well justified with respect to sex and/or gender?** |
| Inclusion criteria required participants to self-identify as MSM, TG, or PWID, be 18 years or older, and be residents of Pakistan. The study excluded sex workers and prisoners due to ethical and legal constraints, which is clearly justified given criminalization and safety risks in Pakistan. The focus on specific groups allows for meaningful investigation of gender-based disparities without compromising participant safety. |
| 1. **Is the data collection method proposed in your study appropriate for investigation of sex and/or gender?** |
| The study used interviewer-facilitated eligibility screening followed by a self-administered digital questionnaire on tablets to protect privacy. Participants could complete the survey in private rooms at CBO sites, ensuring confidentiality, especially for gender minorities. Furthermore, participants were allowed to self-identify their gender from an inclusive list, or opt not to disclose, following ethical guidance on gender data collection. |
| - **Ethics** |
| 1. **Does your study design account for the relevant ethical issues that might have particular significance with respect to gender and/or sex?** |
| The study was carefully designed to account for ethical considerations related to gender, identity, and criminalization, particularly in the context of Pakistan, where same-sex behavior, transgender identity, and drug use are heavily stigmatized and, in some cases, criminalized under laws such as Section 377 of the Pakistan Penal Code and the Narcotic Control Act.  Key ethical considerations that were addressed include:   - Informed consent: All participants gave written informed consent after being verbally informed of the study's purpose, procedures, risks, and their right to withdraw. - Anonymity and privacy: Surveys were self-administered using tablets in private rooms to ensure participants, especially MSM and TG individuals, felt safe and unobserved. - Sensitive recruitment: TG individuals and MSM were recruited via trusted community-based organizations (CBOs), and PWIDs were recruited from rehabilitation centers to reduce security risks and ensure capacity to consent. - Optional gender disclosure: Participants were allowed to self-identify their gender from inclusive options or opt out of disclosing it, aligning with ethical norms for gender-diverse populations. - Protection from harm: The study design deliberately excluded groups such as sex workers and prisoners due to legal risks and ethical challenges in ensuring safe, voluntary participation. |
